# Rosai-Dorfman Disease With Nodal, Nasal, and Laryngeal Involvement: A Case Report

**DOI:** 10.7759/cureus.105866

**Published:** 2026-03-25

**Authors:** Mohamed Afellah, Taha Benatiya Andaloussi, Kaoutar Guemmah, Abdellatif Oudidi

**Affiliations:** 1 Otolaryngology Head and Neck Surgery Department, Centre Hospitalier Universitaire (CHU) Hassan II, Fez, MAR

**Keywords:** airway obstruction, methotrexate therapy, nasopharyngeal involvement, non-langerhans cell histiocytosis, rosai-dorfman disease, tracheostomy

## Abstract

Rosai-Dorfman disease (RDD) is an uncommon histiocytic disorder typically characterized by massive lymphadenopathy and distinctive histopathological findings. Although extranodal involvement is frequently reported, critical airway compromise due to laryngotracheal infiltration remains exceptional.

We describe a 41-year-old woman presenting with systemic disease involving cervical lymph nodes, nasopharynx, nasal cavities, and subglottic airway. Progressive respiratory obstruction led to tracheostomy. Despite initial treatment with systemic corticosteroids and vinblastine, inflammatory activity persisted. Introduction of weekly methotrexate resulted in radiological stabilization and gradual clinical improvement, allowing eventual decannulation.

This case underscores the dual inflammatory and fibrotic mechanisms underlying airway compromise in RDD and highlights the potential role of methotrexate as a long-term immunomodulatory strategy in refractory multisystem involvement.

## Introduction

Rosai-Dorfman disease (RDD) is an uncommon benign histiocytic proliferative disorder first described in 1969 [1]. It is characterized histologically by emperipolesis and immunohistochemical features consistent with non-Langerhans histiocytosis. While nodal involvement is classical, extranodal disease occurs in approximately 30-40% of patients, frequently affecting the head and neck region [2].

ENT involvement may manifest as nasal obstruction, nasopharyngeal mass, laryngeal infiltration, or airway stenosis. Subglottic extension with respiratory compromise is particularly rare and poses significant therapeutic challenges.

We present a case of systemic RDD with progressive pharyngo-laryngeal infiltration and severe airway involvement requiring tracheotomy and long-term immunosuppressive therapy.

## Case presentation

A 41-year-old woman with no significant past medical history initially presented with bilateral cervical lymphadenopathy. She had no known allergies, was not receiving any long-term medications, and had no significant obstetric history. Cervicotomy with histopathological examination revealed non-Langerhans cell hemophagocytic histiocytosis consistent with RDD.

**Figure 1:**
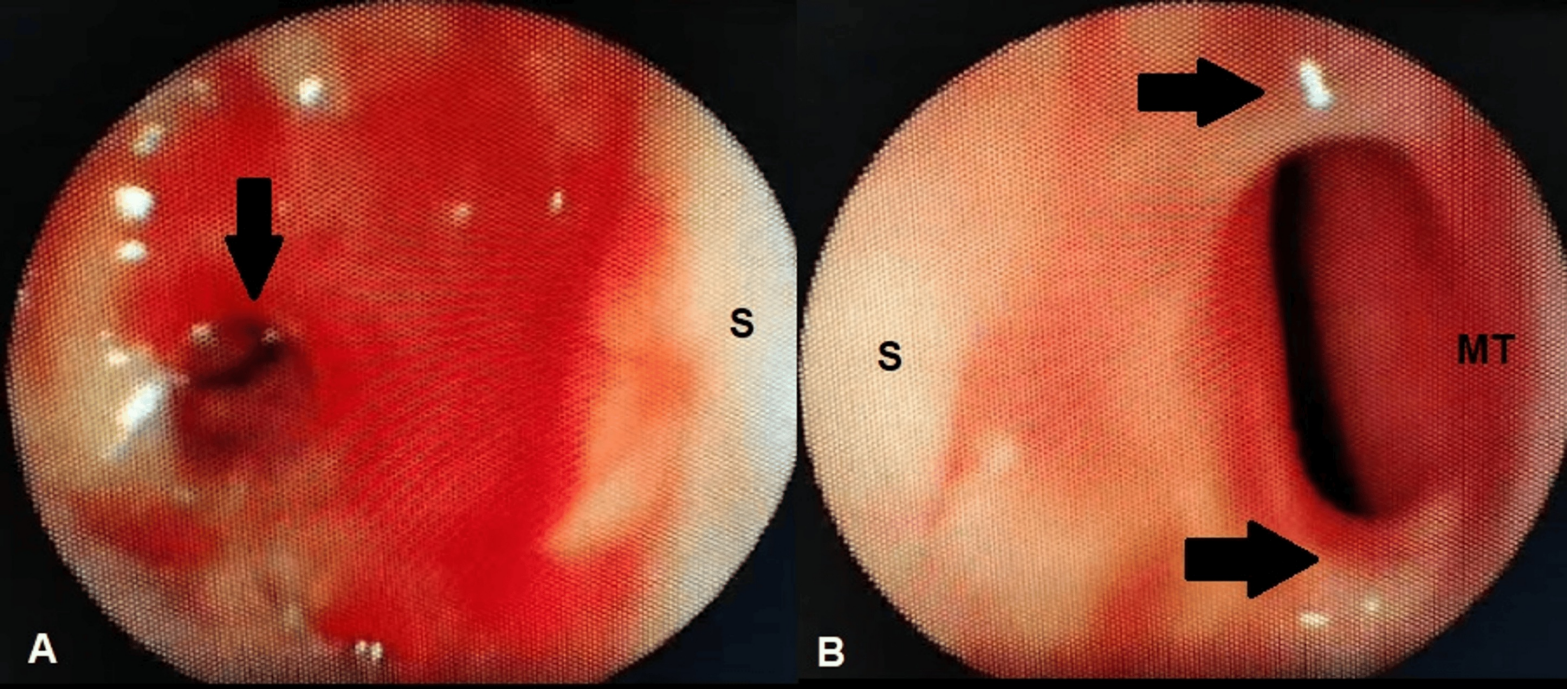
A. Endoscopic view of the right nasal fossa showing choanal synechiae (black arrow) preventing endoscopic passage. B. Endoscopic view of the left nasal fossa showing synechiae between the middle turbinate and the nasal septum (black arrows). S: nasal septum; MT: middle turbinate

A few months later, she developed progressive dyspnea secondary to subglottic stenosis, requiring tracheotomy. Nasofibroscopy demonstrated bilateral nasal synechiae preventing endoscopic passage, bulging of the posterior nasopharyngeal wall covered by intact mucosa, and thickening of the left vocal cord, which remained mobile (Figure 1).

Biopsies of both the subglottic lesion and the nasopharynx revealed mucosa lined by an unremarkable stratified squamous epithelium. The underlying stroma showed a dense, sheet-like inflammatory infiltrate composed predominantly of small lymphocytes admixed with large histiocytes exhibiting emperipolesis, characterized by intact lymphocytes within their cytoplasm. Immunohistochemical analysis demonstrated positivity for S100 and CD68, with negativity for CD1a, supporting the diagnosis of RDD.

**Figure 2:**
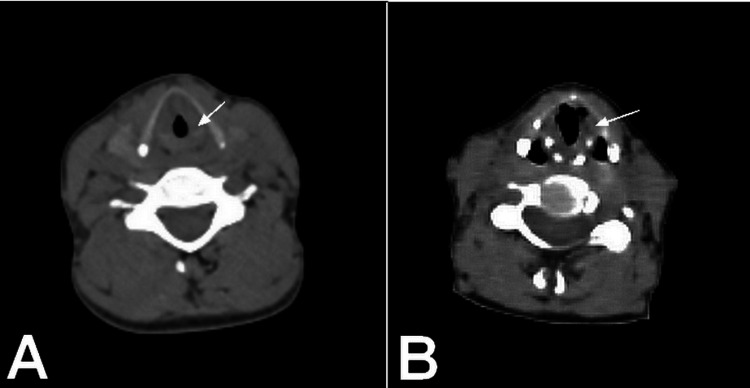
A. Axial CT scan showing a soft-tissue lesion involving the left vocal cord with medial protrusion resulting in partial narrowing of the laryngeal airway (white arrow). B. Follow-up axial CT scan demonstrating marked regression of the lesion after three years of methotrexate therapy, with improvement of the laryngeal airway caliber (white arrow).

Imaging revealed supra- and infra-diaphragmatic lymphadenopathy, along with a left vocal cord soft tissue lesion causing partial narrowing of the laryngeal lumen. Cervicothoracic CT demonstrated persistent homogeneous thickening of the pharyngo-laryngeal tract and middle mediastinal infiltration encasing the trachea and left main bronchus, resulting in significant luminal narrowing (Figure 2).

PET-CT revealed hypermetabolic nasopharyngeal tissue extending to the choanae, nasal cavities, and oropharynx (maximum standardized uptake value (SUVmax) 8.3), as well as retro-tracheal hypermetabolism. Bone scintigraphy revealed isolated right temporal bone hyper-fixation.

Given the progressive active disease, systemic high-dose corticosteroid therapy (prednisolone 2 mg/kg/day) was initiated, followed by nine cycles of vinblastine. Radiological reassessment demonstrated disease stabilization without significant regression. LITAK® therapy was subsequently administered (five cycles), followed by additional vinblastine courses.

Due to refractory disease, methotrexate was introduced at a weekly dose of 12.5-15 mg. Treatment was well-tolerated with regular hepatic, hematologic, and renal monitoring, and laboratory parameters remained stable throughout follow-up.

**Figure 3:**
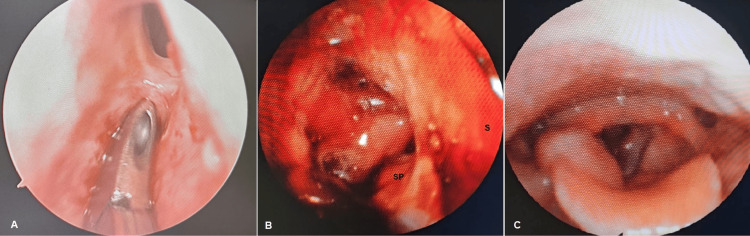
A. Endoscopic view showing release of the synechiae between the middle turbinate and the nasal septum. B. Endoscopic view showing release of choanal synechiae with visualization of the soft palate. C. Endoscopic view demonstrating adequate laryngeal airway patency. SP: soft palate; S: nasal septum

Surgical release of the nasal synechiae was performed with bilateral nasal packing and postoperative placement of silastic splints (Figure 3A). Follow-up nasofibroscopy demonstrated adequate restoration of nasal patency (Figure 3B). In the context of clinical stabilization under methotrexate, progressive improvement in airway status allowed successful decannulation and closure of the tracheotomy site (Figure 3C).

This case highlights that, although corticosteroids may control inflammatory activity in RDD, laryngotracheal involvement can result in persistent structural airway damage. Methotrexate may achieve disease stabilization in refractory cases; however, reversal of established fibrotic airway stenosis remains limited, underscoring the importance of early intervention before irreversible remodeling occurs.

The chronological progression of clinical events, investigations, and therapeutic interventions is summarized in Table 1.

**Table 1:** Timeline of clinical events and management

Year	Clinical Event	Investigation	Treatment
2017	Acute laryngeal dyspnea	Nasofibroscopy: obstructive laryngeal mass	Emergency tracheostomy
2017	Diagnostic workup	Panendoscopy + biopsies (nasal, laryngeal, lymph node)	—
2017	Diagnosis confirmed	Histology + immunohistochemistry	—
2017–2018	Active disease	Imaging (CT scan)	High-dose corticosteroids
2018–2020	Persistent disease	Follow-up imaging	Vinblastine cycles
2021	Refractory disease	Clinical + radiological evaluation	Methotrexate initiated
2021–2025	Stabilization	Serial imaging	Methotrexate maintenance
2025	ENT reassessment	Nasofibroscopy: synechiae	Decannulation + synechiae lysis

## Discussion

RDD is a rare, benign, non-Langerhans cell histiocytosis first described in 1969 [1]. Extranodal involvement occurs in approximately 30-40% of cases [2] and is characterized by the accumulation of activated histiocytes. Although viral and immune dysregulation hypotheses have been proposed, no definitive pathogenic mechanism has been established [3]. While nodal disease is classical, extranodal involvement frequently affects the head and neck region. However, clinically significant laryngotracheal obstruction remains uncommon and poorly described in the literature.

Airway compromise

The upper airway manifestations of RDD typically present as nasal obstruction, sinonasal masses, or nasopharyngeal infiltration. Laryngeal involvement is considerably rarer and may manifest as dysphonia, dyspnea, or progressive airway narrowing [4]. Subglottic extension leading to critical stenosis requiring tracheostomy is exceptional.

In our patient, progressive subglottic narrowing resulted in severe dyspnea requiring emergency tracheostomy. Radiological assessment demonstrated circumferential thickening of the pharyngolaryngeal tract, with associated mediastinal infiltration encasing the trachea and left main bronchus. These findings suggest two complementary mechanisms of airway compromise in RDD: direct inflammatory infiltration of the laryngeal and subglottic mucosa, and extrinsic compression secondary to mediastinal soft tissue involvement. The coexistence of these processes likely contributed to the persistence of airway narrowing despite systemic therapy.

Unlike purely inflammatory lesions that regress with corticosteroid therapy, chronic RDD lesions may evolve toward fibrotic remodeling. Once fibrosis develops, structural airway damage may become irreversible, explaining the prolonged tracheostomy dependence observed in our patient.

Nasal and nasopharyngeal involvement

Sinonasal involvement is among the most common extranodal manifestations of RDD. Our patient exhibited bilateral nasal synechiae and nasopharyngeal infiltration confirmed by PET-CT hypermetabolism. The development of synechiae likely reflects chronic inflammatory mucosal injury followed by fibrotic healing. Surgical release was required to restore nasal patency, underscoring that systemic disease control does not necessarily prevent local fibrotic sequelae in the ENT region.

Therapeutic considerations

There is no standardized treatment protocol for RDD due to its rarity and heterogeneous presentation. According to expert consensus recommendations from the Histiocyte Society, management should be individualized based on disease severity and organ involvement [5].

Systemic corticosteroids remain first-line therapy for symptomatic or organ-threatening RDD due to their rapid anti-inflammatory effects [5]. In our patient, corticosteroids were initiated during the active phase; however, the response was incomplete. Vinblastine has been used in disseminated or refractory RDD with variable outcomes [5]; in this case, multiple cycles resulted in disease stabilization rather than regression.

For refractory or multisystem disease, immunomodulatory agents, including methotrexate, azathioprine, 6-mercaptopurine, and thalidomide, have been reported with varying success [5]. Methotrexate was subsequently introduced at a weekly dose of 12.5 mg as a steroid-sparing agent. It exerts anti-inflammatory effects through the inhibition of dihydrofolate reductase and modulation of cytokine pathways [6]. Although its role in RDD is not well-established, it has been reported in refractory multisystem disease.

Only a limited number of cases of laryngeal or subglottic RDD have been reported. In most cases, airway involvement was managed with corticosteroids and/or surgical intervention, with variable outcomes. Compared to these reports, our case is distinguished by the severity of airway obstruction requiring tracheostomy and by the favorable long-term outcome, with successful decannulation under methotrexate therapy.

In this patient, methotrexate resulted in radiological stabilization and partial regression of pulmonary inflammatory lesions, although established airway fibrosis persisted. This suggests that methotrexate may effectively control inflammatory activity but cannot reverse structural sequelae once fibrosis has occurred.

Lessons from this case

This case highlights several important clinical considerations: RDD should be considered in unexplained subglottic stenosis associated with lymphadenopathy; Airway involvement may be multifactorial, combining intrinsic infiltration and extrinsic compression; Early immunosuppressive therapy may limit inflammatory progression; however, delayed diagnosis may result in irreversible fibrotic damage; Methotrexate appears to be a viable long-term therapeutic option in refractory multisystem RDD; Although methotrexate use in RDD has been previously reported, this case further illustrates its role in long-term disease stabilization in the setting of severe airway involvement and delayed decannulation.

Future studies involving larger cohorts and diverse populations are needed to better characterize the full spectrum of extranodal involvement, particularly in sites such as the skin, bone, and central nervous system.

## Conclusions

This case report aimed to highlight the diagnostic and therapeutic challenges of Rosai-Dorfman disease with severe otorhinolaryngological involvement, particularly in the context of life-threatening airway obstruction. It illustrates that laryngeal RDD, although rare, should be considered in cases of unexplained upper airway obstruction associated with lymphadenopathy. Our observation emphasizes that early recognition and prompt management are crucial to prevent irreversible airway damage. While high-dose corticosteroids can control the inflammatory phase, methotrexate appears to be an effective long-term therapeutic option for disease stabilization in refractory cases. However, once fibrotic remodeling is established, structural airway sequelae may persist despite adequate systemic treatment.

This case underscores the importance of a multidisciplinary approach combining internal medicine and otorhinolaryngology to optimize both functional and systemic outcomes.
